# Amino Acid Transporters on the Guard of Cell Genome and Epigenome

**DOI:** 10.3390/cancers13010125

**Published:** 2021-01-02

**Authors:** Uğur Kahya, Ayşe Sedef Köseer, Anna Dubrovska

**Affiliations:** 1 OncoRay–National Center for Radiation Research in Oncology, Faculty of Medicine and University Hospital Carl Gustav Carus, Technische Universität Dresden, Helmholtz-Zentrum Dresden-Rossendorf, 01309 Dresden, Germany; Ugur.Kahya@uniklinikum-dresden.de (U.K.); AyseSedef.Koeseer@uniklinikum-dresden.de (A.S.K.); 2 Helmholtz-Zentrum Dresden-Rossendorf, Institute of Radiooncology-OncoRay, 01328 Dresden, Germany; 3 National Center for Tumor Diseases (NCT), Partner Site Dresden and German Cancer Research Center (DKFZ), 69120 Heidelberg, Germany; 4 Faculty of Medicine and University Hospital Carl Gustav Carus, Technische Universität Dresden, 01307 Dresden, Germany; 5 German Cancer Consortium (DKTK), Partner Site Dresden and German Cancer Research Center (DKFZ), 69120 Heidelberg, Germany

**Keywords:** amino acid transporters, oxidative stress, reactive oxygen species (ROS), glutathione (GSH), α-ketoglutarate (αKG), SLC7A11/xCT, SLC7A5/LAT1, SLC1A5/ASCT2, epigenetic regulation, Enhancer of zeste homolog 2 (EZH2)

## Abstract

**Simple Summary:**

Amino acid transporters play a multifaceted role in tumor initiation, progression, and therapy resistance. They are critical to cover the high energetic and biosynthetic needs of fast-growing tumors associated with increased proliferation rates and nutrient-poor environments. Many amino acid transporters are highly expressed in tumors compared to the adjacent normal tissues, and their expression correlates with tumor progression, clinical outcome, and treatment resistance. Tumor growth is driven by epigenetic and metabolic reprogramming and is associated with excessive production of reactive oxygen species causing the damage of vital macromolecules, including DNA. This review describes the role of the amino acid transporters in maintaining tumor redox homeostasis, DNA integrity, and epigenetic landscape under stress conditions and discusses them as potential targets for tumor imaging and treatment.

**Abstract:**

Tumorigenesis is driven by metabolic reprogramming. Oncogenic mutations and epigenetic alterations that cause metabolic rewiring may also upregulate the reactive oxygen species (ROS). Precise regulation of the intracellular ROS levels is critical for tumor cell growth and survival. High ROS production leads to the damage of vital macromolecules, such as DNA, proteins, and lipids, causing genomic instability and further tumor evolution. One of the hallmarks of cancer metabolism is deregulated amino acid uptake. In fast-growing tumors, amino acids are not only the source of energy and building intermediates but also critical regulators of redox homeostasis. Amino acid uptake regulates the intracellular glutathione (GSH) levels, endoplasmic reticulum stress, unfolded protein response signaling, mTOR-mediated antioxidant defense, and epigenetic adaptations of tumor cells to oxidative stress. This review summarizes the role of amino acid transporters as the defender of tumor antioxidant system and genome integrity and discusses them as promising therapeutic targets and tumor imaging tools.

## 1. Introduction

Cancer is a metabolic disease, and tumor development is driven by metabolic reprogramming [1,2]. Tumorigenesis is associated with the accumulation of genetic mutations and epigenetic alterations that drive cancer cell proliferation and increase tumor nutrient demands. Tumor cells must increase the uptake of glucose and amino acids to cover the energetic and biosynthetic needs associated with high proliferation rates and environmental changes. An increased rate of glucose consumption by tumor cells, even in the presence of oxygen (aerobic glycolysis), was first described in the 1920s by Otto Warburg [3]. Later, elevated glucose uptake has been confirmed for a wide variety of tumor types and employed to develop fluorodeoxyglucose-based (^18^F)-positron emission tomography (^18^F-FDG PET), a clinical tumor imaging for diagnostics, staging, and analysis of treatment response. Although ^18^F-FDG PET is commonly used for the evaluation of cancer patients, it cannot be applied to all tumor entities; thus, alternative precise and accurate imaging approaches are being established based on the amino acid consumption and high expression levels of the amino acid transporters in tumor cells. The high demand of tumor cells for amino acid glutamine (Gln) was first highlighted in the 1950s by Harry Eagle. He noticed that HeLa cells have about ten-fold increased Gln consumption compared to fibroblast culture and cannot survive without Gln supplementation [4]. Although Gln is not considered an essential amino acid (EAA), many tumor types are Gln-addicted and need to acquire Gln from external sources to grow [5,6]. In addition to glucose, Gln is a principal nutrient in fast-growing tumors serving as a source of energy and building intermediates to synthesize critical macromolecules such as DNA and proteins [7]. Gln also regulates the uptake of EAAs, which cannot be produced de novo, by antiport mechanisms [8].

Metabolic reprogramming driven by hypoxia and tumor-associated genetic mutations can induce excessive production of reactive oxygen species (ROS) in cancer cells. Consequently, high tumor-produced ROS levels affect the metabolic and proliferative properties of cancer-associated fibroblasts and inhibit the anti-tumor immune response [9,10]. ROS are mainly generated by the electron transport chain in mitochondria, upon oxidative protein folding in the endoplasmic reticulum (ER) and during some enzymatic reactions involving, e.g., cyclooxygenases, lipoxygenases, xanthine oxidases, and nicotinamide-adenine dinucleotide phosphate (NADPH) oxidases [11,12,13,14]. At fine-tuned levels, ROS-mediated redox signaling is essential for the regulation of many cellular processes in normal tissues, including cell proliferation, growth, migration, and differentiation, as reviewed elsewhere [15,16]. Although ROS play an essential role as intracellular second messengers, increased, ROS production causes the damage of vital macromolecules, including DNA mutations, protein oxidation, and lipid peroxidation [11,12,13,14]. ROS-dependent DNA damage and dysregulation of intracellular signaling such as upregulation of hypoxia response genes may lead to genomic instability, carcinogenesis, and the development of other pathologies. 

To counter the harmful effect of high ROS levels, cells are equipped with ROS scavenging molecules such as glutathione (GSH) and thioredoxins, as well as ROS catabolic enzymes such as glutathione peroxidase (GPX), catalase (CAT), and superoxide dismutase (SOD) to overcome the increased ROS production.

**Figure 1:**
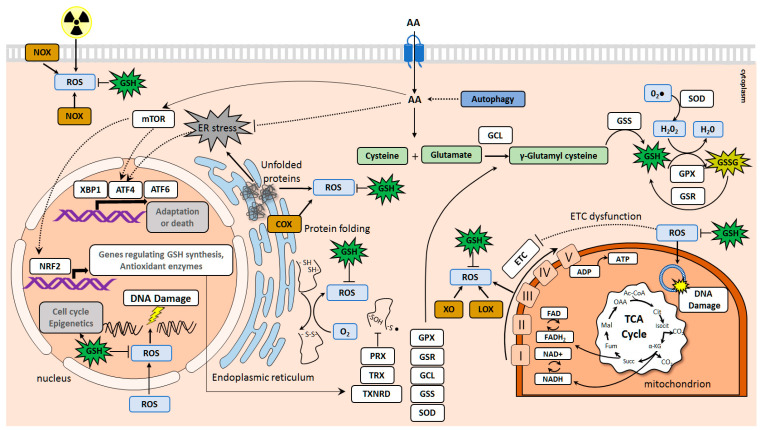
Amino acid transporters in the maintenance of redox homeostasis. The reactive oxygen species (ROS) are generated in the cells in different ways, including the electron transport chain (ETC) in mitochondria, oxidative protein folding in the endoplasmic reticulum (ER), and enzymatic reactions involving, e.g., cyclooxygenases (COX), lipoxygenases (LOX), xanthine oxidases (XO), and nicotinamide adenine dinucleotide phosphate (NADPH) oxidases (NOX). An increased ROS production contributes to the damaging of DNA, lipids, and proteins. Amino acid transporters regulate the redox homeostasis by driving glutathione (GSH) synthesis, inducing mammalian target of rapamycin (mTOR)-mediated antioxidant defense, and preventing endoplasmic reticulum (ER) stress. Autophagy also contributes to the maintenance of the intracellular free amino acid pool. GSH is synthesized in cytosol from Cys and Glu by glutamate-cysteine ligase (GCL) and glutathione synthetase (GSS). The reduced form of glutathione, GSH, is oxidized to glutathione disulfide (GSSG) by glutathione peroxidase (GPX) to neutralize hydrogen peroxide (H_2_0_2_) and is reduced back to GSH by glutathione reductase (GSR). The mTOR mechanism of antioxidant defense includes activation of the nuclear factor-erythroid 2 -related factor 2 (NRF2), a master transcription factor. NRF2 regulates the expression of many genes involved in the GSH synthesis, including *GCL*, *GSS*, *GPX*, *GSR,* other genes involved in the regulation of ROS levels, such as superoxide dismutase (*SOD*) enzyme which converts superoxide (O_2_^•−^) to H_2_0_2_, genes encoding thiol-specific antioxidant proteins thioredoxin (TRX) and thioredoxin reductase (TXNRD) as well as peroxiredoxin (PRX). mTOR also triggers ATF4-mediated gene expression of the heterodimeric transmembrane amino acid transporters such as *SLC7A5* (*LAT1*), *SLC3A2* (*CD98hc*), and *SLC7A11* (*xCT*).

The reduced form of GSH is an essential free radical scavenger that plays a critical role in cancer progression and treatment resistance [17]. GSH is synthesized in cytosol from cysteine (Cys) and glutamate (Glu) and then distributed into different organelles, including mitochondria, ER, and nucleus. Intracellular GSH levels can be regulated by modulating the uptake of the precursor amino acids, including Cys, Gln, Glu, and glycine (Gly), by specific amino acid transporters. Gln is converted in the cytosol to Glu by glutaminase (GLS). The reduced form of GSH is oxidized to glutathione disulfide (GSSG) by GPX to convert hydrogen peroxide (H_2_0_2_) into water. GSSG is reduced back to GSH by glutathione reductase (GSR) [18]. GSH levels were found to be high in many tumor types that have important implications for patient survival [19,20]. Increased antioxidant capacity of tumor cells forces their resistance to the prooxidative radio- and chemotherapy [20,21,22]. Amino acid availability contributes to the maintenance of the redox homeostasis by regulating the mammalian target of rapamycin (mTOR)-mediated antioxidant defense, ER stress, and unfolded protein response (UPR) signaling [23,24] (Figure 1). Furthermore, recent findings suggest epigenetic mechanisms to overcome oxidative stress triggered by amino acid uptake and amino acid transporter expression. Amino acid transporters are upregulated in the different types of cancer, and many of them, such as CD98hc/SCL3A2, LAT1/SLC7A5, ASCT2/SLC1A5, emerged as promising molecular biomarkers, therapeutic targets, and novel tools for tumor imaging. 

This review summarizes the multifaceted role of the amino acid transporters in the maintenance of tumor redox homeostasis, DNA integrity, and epigenetic landscape under stress conditions and discusses them as potential targets for tumor imaging and treatment.

## 2. Amino Acid Transporters Upregulated in Tumors and Their Role in Cell Protection against Oxidative Damage and DNA Injury

**Figure 2:**
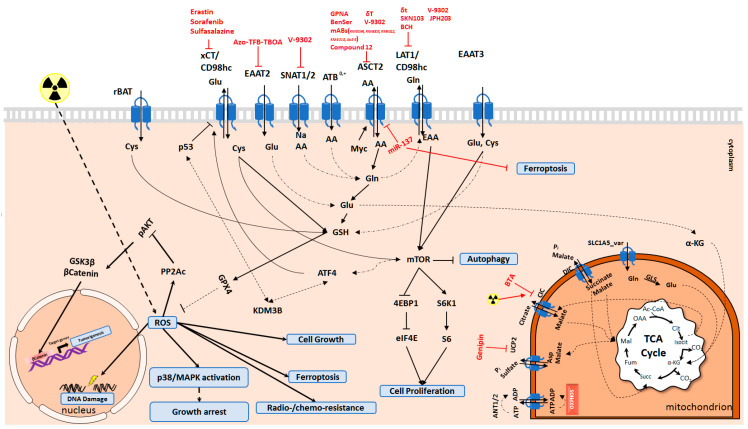
Molecular mechanisms activated by amino acid transporters. Amino acid transporters are vital to balance the intracellular amino acid pool for many cellular functions. Depending on the amino acid transporter activity, ferroptosis, apoptosis, autophagy, and proliferation of cells is regulated. Cys and Glu are the key amino acids for the synthesis of GSH. Cells deal with ROS species through GSH to decrease DNA damage, protein oxidation, and lipid peroxidation. GLS: Glutaminase; GSH: Glutathione; Cys: Cysteine; Glu: Glutamate; Gln: Glutamine; Asp: Aspartate; AA: Amino acids; EAA: Essential amino acids; αKG: alpha-ketoglutarate.

Expression of the amino acid transporters is upregulated in many tumor malignancies and enhances tumor aggressiveness, drug resistance, and oxidative damage protection. Hence, targeting amino acid transporters as an Achilles’ heel of the cancer cells may offer a wide range of opportunities to tackle oxidative stress and sensitize tumor cells to anticancer treatments (Figure 2). 

### 2.1. SLC1 Family of Amino Acid Transporters

The Solute Carrier 1 (SLC1) family of amino acid transporters is comprised of seven members, which are essential not only to balance extracellular glutamate concentration under neurotoxic levels in the central nervous system but also contribute to the inter-organ Gln flux and nitrogen metabolism in peripheral tissues [25]. While five members of this family (SLC1A1, SLC1A2, SLC1A3, SLC1A6, and SLC1A7) function as high-affinity Glu transporters, the remaining two (SLC1A4 and SLC1A5) work as neutral amino acid transporters [25]. In general, members of this family are essential for activation of the mTOR pathway to maintain cell growth and proliferation as reviewed in [26], and for coping with oxidative stress due to their contribution to the synthesis of GSH [27,28,29].

Excitatory amino acid transporter 3 (EAAT3/SLC1A1) is a critical regulator of GSH synthesis and oxidative stress in neurons [30,31] and an important contributor to tumorigenesis [32]. SLC1A1 expression, which is essential for Glu and Cys import, is sustained by Sortilin Related VPS10 Domain Containing Receptor 2 (SorCS2) [30]. The shortage of SorCS2 leads to the decreased surface expression of SLC1A1 due to its targeting to the late endosomes and results in oxidative brain damage and enhanced neuronal cell death [30]. The serine/threonine mTOR kinase strongly upregulates SLC1A1 expression [33]. Reciprocally, a high level of SLC1A1 augments expression of activating transcription factor 4 (ATF4) and Cys/Glu antiporter xCT/SLC7A11 as well as phosphorylation of mTOR, the mTOR Substrate S6 Kinase 1 (S6K1), and eukaryotic translation initiation factor 4E-binding protein 1 (4EBP1) [28]. Henceforth, SLC1A1 may activate the mTOR pathway to elevate intracellular Cys levels through increased ATF4 and SLC7A11 expression to cope with the oxidative stress [28].

Alanine, serine, cysteine-preferring transporter 2 (ASCT2), encoded by SLC1A5 in humans, is a member of SLC family proteins that mediates uptake of Gln, which becomes a conditional EAA in the highly proliferative cancer cells [5,6] and supports intracellular amino acid homeostasis maintaining the activity of mTOR signaling pathway [6,34]. SLC1A5 expression is regulated by several transcription factors such as MYC, retinoblastoma/E2F transcription factor pathway (RB/E2F), androgen receptor (AR), and ATF4 [35,36,37,38,39,40]. SLC1A5 depletion leads to an amino acid starvation response in 143B osteosarcoma cells. Depletion of SLC1A5 expression upregulates sodium neutral amino acid transporters from the SLC38 family, sodium-coupled neutral amino acid transporter SNAT1/ SLC38A1 and SNAT2/SLC38A2 to compensate for the lack of SLC1A5 due to their similarity in substrate specificity [34,41,42]. Furthermore, Gln metabolism is promoted by the AR signaling, which upregulates SLC1A4/ASCT1 and SLC1A5 expression to maintain cell growth under serum starvation conditions via mTOR signaling [27]. Consistent with this observation, SLC1A5 knockdown reduces androgen-mediated Gln uptake and growth of androgen-sensitive prostate cancer (PCa) cell lines [27]. Also, a variant of SLC1A5, called SLC1A5_var, promotes metabolic switch by augmenting Gln metabolism in cancer cells [43]. Unlike SLC1A5, SLC1A5_var is localized to the mitochondrial membrane because of its N-terminal mitochondrial targeting signal and may mediate the Gln influx in mitochondrial respiration. The expression of SLC1A5_var is triggered by hypoxia and plays an essential role in the induction of the oxidative phosphorylation (OXPHOS) and increased ROS scavenging attributed to the Gln-derived GSH synthesis [43].

lncRNA X-inactive specific transcript (lncRNA-XIST) is a long non-coding RNA that promotes glioblastoma progression by sponging microRNA (miRNA) miR-137, which targets SLC1A5 expression. Knockdown of lncRNA-XIST reduces SLC1A5 levels and inhibits tumorigenesis of glioma cells in vivo [44]. Correspondingly, lncRNA-XIST knockdown in glioma promotes ROS production and apoptosis and inhibits cell proliferation in SLC1A5/miR-137 dependent fashion, which can be reversed by cell supplementation with N-acetyl-L-Cysteine (NAC), a scavenger of ROS [44]. miR-137 also reduces Gln uptake by directly targeting SLC1A5 in melanoma cells and triggers ROS generation and ferroptosis, a form of the non-apoptotic, iron-dependent mode of cell death associated with the accretion of lipid ROS [45]. Normally, GSH decreases reactive oxygen and nitrogen species (RONS) through glutathione peroxidase (GPX) activity. On the contrary, as a result of a reduction in the intracellular GSH levels and glutathione peroxidase 4 (GPX4) activity, lipid peroxidation cannot be controlled anymore, leading to increased ROS levels and cell death [45]. All in all, these findings suggest that targeting SLC1A5 might offer a new therapeutic strategy for the treatment of different types of malignancies [46].

### 2.2. SLC3 and SLC7 Families of Heteromeric Amino Acid Transporters

SLC7 family is composed of L-type amino acid transporters (LATs, SLC7A5-13, and SLC7A15) and cationic amino acid transporters (SLC7A1-4, and SLC7A14) [47]. LATs are the light subunits of the heterodimeric transmembrane amino acid transporter complexes (HATC) forming the heterodimers with the heavy subunits of SLC3 family: SLC3A1 and SLC3A2 [47]. In general, members of SLC3 and SLC7 families are essential for mTOR pathway activation as reviewed in [26], and for the regulation of autophagy and ferroptosis by balancing intracellular AA and ROS levels [48,49,50,51,52,53,54].

rBAT is encoded by SLC3A1 and is overexpressed in breast cancer cells promoting tumorigenesis via rBAT mediated Cys uptake [48]. Elevated Cys uptake reduced the activity and stability of serine/threonine-protein phosphatase 2A catalytic subunit alpha isoform (PP2Ac) via ROS level reduction; therefore, it induces activation of AKT signaling [48]. 

CD98hc/SLC3A2 acts as a part of the transporter complexes for the branched-chain amino acids (BCAA) and aromatic amino acids (AAA). It serves as a regulator of mTOR signaling and enhancer of integrin signaling. The amino acids transported by SLC3A2 HATCs are essential for synthesizing purine and pyrimidine nucleotides and cell cycle progression. The SLC3A2 depletion results in the critical decrease of nucleotide availability and induces replicative stress [55]. SLC3A2 functions as a chaperon for the light subunits and is important for their stability and membrane localization [49,56,57,58,59]. SLC3A2 can form HATCs with xCT (encoded by SLC7A11), LAT1 (encoded by SLC7A5), and y+LAT2 (encoded by SLC7A6). 

The depletion of SLC3A2 HATCs results in a reduced intracellular pool of amino acids, including leucine (Leu) and arginine (Arg), potent activators of the mTOR kinase [59,60]. The intracellular amino acid deficiency results in the mTOR signaling inhibition and induces activation of general amino acid control non-derepressible 2 (GCN2), which phosphorylates the subunit of eukaryotic translation initiation factor 2A (eIF2α). These mechanisms repress protein synthesis and trigger the expression of ATF4, which regulates the transcription of multiple targets involved in amino acid metabolism and stress response [61,62]. In particular, ATF4 is essential for the expression of SLC7A5, SLC7A11, and SLC3A2 and, therefore, for the replenishment of the intracellular GSH precursors [51,62,63,64,65]. The ATF4 knockout (KO) cells possess an impaired amino acid import and GSH biosynthesis and are highly predisposed to oxidative stress [62]. The adaptive signaling pathway triggered by ATF4 to recover cellular homeostasis is called the integrated stress response (ISR). ISR leads to the inhibition of the global mRNA translation and is one of the key cell survival strategies in the face of different stresses, including ER stress. The ER is an organelle responsible for protein folding and degradation. Hydrogen peroxide is formed as a byproduct of the disulfide bond formation [66]. The ER consumes cellular GSH that buffers the ER against these hyperoxidizing conditions [67]. The ER stress is induced by the accumulation of unfolded proteins and the formation of improper disulfide bonds. During this process, the released electrons transferred to molecular oxygen result in ROS accumulation as a byproduct and lead to the depleting of the intracellular GSH pool [67,68]. The accumulation of unfolded proteins in ER triggers UPR signaling, which might serve either as a pro-survival or pro-apoptotic process. The exact mechanisms determining the cell fate in response to UPR remains to be yet investigated, although it has been suggested that mitochondrial respiration substantially contributes to the lethal ROS accumulation during ER stress and UPR activation [68,69]. SLC3A2 plays a role in ER stress-related UPR as knockdown of SLC3A2 expression inhibited expression of three key UPR proteins ATF4, ATF6, and XBP1, indicating that the ER stress-mediated UPR is interrupted as a result of SLC3A2 inhibition [70].

The ER stress and UPR - mediated activation of the nuclear factor kappa B (NF-κB) and p38/MAPK (mitogen-activated protein kinase) signaling results in the inhibition of Akt/mTOR/p70S6K pathway and activation of autophagy [71,72,73]. Autophagy plays a dual role as a mechanism that either induces or inhibits cell death depending on the tissue context and environmental cues [74]. Pro-survival autophagy promotes the removal and degradation of unfolded proteins and damaged organelles while providing starved cells with amino acids for protein biosynthesis and energy replenishment [75].

Downregulation of SLC3A2 expression results in depletion of the reduced form of GSH, increased oxidative stress, DNA damage, and radiosensitivity in head and neck squamous carcinoma (HNSCC) cells [49]. Consequently, high expression of SLC3A2 and SLC7A5 contributes to the increased radioresistance in HNSCC in vitro and correlates with a worse outcome in patients with HNSCC treated with radiochemotherapy. Simultaneously, SLC3A2 depletion increases pro-survival autophagy in HNSCC, which helps tumor cells to survive on nutrient shortage and stress conditions. Inhibition of autophagy with bafilomycin A treatment or ATG5 knockdown sensitizes SLC3A2 KO HNSCC cells to ionizing radiation (IR).

SLC7A11 is a subunit of the glutamate/cystine antiporter system xc-. The SLC3A2/ SLC7A11 HATC is essential for the transport of cystine, the oxidized form of Cys, and thus for GSH biosynthesis and the maintaining of cellular redox homeostasis [56,76]. Oxidative stress conditions induce SLC7A11 mRNA expression mediated by transcription factor Nuclear Factor-Erythroid 2-related factor 2 (NRF2), and ATF4. NRF2 and ATF4 bind to the antioxidant response element (ARE) and amino acid response element (AARE), respectively, in the promoter regions of *SLC7A11* gene [63,77]. xCT encoded by *SLC7A11* is essential for cancer cell survival and tumor formation in vivo. The upregulation of SLC7A11 expression by oncogenic KRAS pathway has been shown to contribute to RAS transformation via regulation of redox balance [64,78,79]. The genetic disruption or chemical inhibition of SLC3A2/ SLC7A11 HATC leads to autophagy and ferroptosis in the different types of tumor cells, including endometrial, colorectal, pancreatic cancer, and hepatocellular carcinoma (HCC) through inhibition of mTOR/p70S6K signaling [45,51,56,57,78,80,81]. On the other hand, SLC7A11 is reciprocally regulated by the mTOR pathway. Mammalian target of rapamycin complex 2 (mTORC2) phosphorylates cytosolic N terminus of SLC7A11 at Serine26 (Ser26) and therefore inhibits its activity [52]. mTOR also induces the phosphorylation of eukaryotic initiation factor 2 alpha (eIF2a). eIF2a triggers ATF4 translation and ATF4-mediated gene expression to maintain the supply of amino acids for GSH and protein biosynthesis [62,82]. These ATF4 target genes include the members of SLC3A2 HATCs such as *SLC7A5* (*LAT1*), *SLC3A2* (*CD98hc*) and *SLC7A11* (*xCT*) [51,62,63,64,65,83]. 

Mitochondrial dysfunction associated with DNA mutations activates a retrograde mitochondria-to-nucleus response inducing stress defense and metabolic reprogramming [84]. This retrograde signaling contributed to mitochondrial dysfunction-induced cisplatin resistance in a gastric cancer model through activation of the eIF2α-ATF4 pathway and consequent induction of SLC7A11 expression [85]. Pharmacological inhibition of SLC7A11 with sulfasalazine (SSZ) or erastin, genetic silencing of SLC7A11 expression, or inhibition of GSH synthesis with buthionine sulphoximine (BSO) restores cisplatin sensitivity in gastric cancer cells [85]. Moreover, SLC7A11 expression is essential for the anti-cancer toxicity of temozolomide (TMZ), a DNA alkylating agent used to treat malignant gliomas. Tumor cells with SLC7A11 knockdown exhibit higher vulnerability towards TMZ treatment and SLC7A11 inhibitors such as erastin and sorafenib potentiate a toxic impact of TMZ by induction of ferroptosis [86]. Reduction of the intracellular GSH in response to SLC7A11 inhibition with erastin sensitizes breast cancer cells to γ-radiation by induction of complex DNA damage [87].

Therapy resistance has been attributed to the populations of the tumor-initiating cells called cancer stem cells (CSCs) identified in most tumor entities [88,89,90]. A cell surface adhesion protein Cluster of Differenation 44 (CD44) was characterized as one of the CSC markers for different tumors [91]. A CD44 variant (CD44v) interacts with and stabilizes SLC7A11 at the plasma membrane, and therefore positively regulates the GSH levels and ROS scavenging in tumor cells, and in particular, in CD44-expressing colorectal and gastrointestinal CSCs [92,93]. Activation of the ROS scavenging system in CSCs is associated with chemo- and radioresistance [94,95], and targeting SLC7A11 may be a promising approach to eliminate therapy-resistant CSCs [92,93].

The ubiquitin-proteasome system (UPS) is one of the major ways to degrade and remove the oxidatively damaged and misfolded proteins and recycle the resulting amino acids. The disruption of this pathway by proteasome inhibitors induces ER stress and ROS production [96,97]. Proteasome activity is elevated in many human cancers, and CSCs are shown to be highly sensitive to proteasome inhibition [98,99]. Proteasome inhibition with bortezomib induces SLC7A11 expression by the ATF4 and NRF2-dependent mechanisms to increase the intracellular Cys pool and GSH biosynthesis. Genetic depletion of SLC7A11 or pharmacological inhibition of its function increases tumor cell sensitivity to the proteasome inhibition [63].

Although downregulation of SLC7A11 inhibits GSH synthesis and induces oxidative stress, it leads to the increased expression of the efflux transporter P-glycoprotein (P-gp), also called multidrug resistance protein 1, MDR1, and therefore induces a drug-resistant phenotype in breast cancer cells. The study by Ge et al. suggests that reversing the P-gp overexpression and potential collateral sensitivity of P-gp expressing cells to certain chemical drugs might be utilized to increase the anti-cancer potential of the SLC7A11-targeting agents [100,101].

### 2.3. SLC38 Family of Sodium-Dependent Neutral Amino Acid Transporters

SLC38 family includes 11 Na+-dependent transporters of neutral amino acids [102]. The activity of SLC38 family members is pH-dependent and notably elevated at alkaline pH conditions [102]. SNAT2/SLC38A2 is a rescue amino acid transporter responsible for the Gln uptake under stress conditions [42]. Amino acid deprivation triggers an integrated stress response by cyclin-dependent kinase 7 (CDK7)-mediated SLC38A2 upregulation [103]. Besides, SLC38A2 expression is induced by hypoxia and estrogen receptor alpha (ERα) and contributes to the resistance to anti-endocrine therapy in breast cancer [104]. High SLC38A2 expression is associated with poor clinical prognosis in breast cancer patients, including triple-negative breast cancer (TNBC) [104]. TNBC cells highly depend on Gln uptake to support Cys transport by the SLC7A11 antiporter [105]. SLC38A2 knockdown reduces Gln transport and increases ROS levels upon Gln deprivation [104]. SLC38A3/SNAT3 transports Gln, histidine (His), and aspartate (Asp) [106]. SLC38A3 and NRF2 expression in the kidney tissues is elevated during metabolic acidosis [107]. NRF2 directly induces SLC38A3 mRNA expression upon metabolic acidosis to increase Gln influx and maintain pH homeostasis. Besides, the Nrf2 KO mice exhibited acidosis-induced enhancement of oxidative stress and failed to enhance Slc38a3 expression [107].

### 2.4. SLC25 Family of Mitochondrial Transporters

SLC25 is the largest family of solute transporters in humans. It includes 53 members and is composed of 24 evolutionary well-conserved subfamilies. Most SLC25 transporters function as monomers and are responsible for the transport of their substrates across the inner mitochondria membrane [108,109]. These transporters function as a bridge between metabolic reactions in mitochondria and cytosol by transporting a wide variety of solutes, including nucleotides, amino acids, vitamins, inorganic ions, fatty acids, dicarboxylate, and citrate. The defects of mitochondrial carriers are associated with dysregulation of the mitochondrial functions and cellular metabolism [108,109]. SLC25 transporters were also characterized as regulators of intracellular ROS levels. The dicarboxylate carrier, DIC encoded by the *SLC25A10* gene, regulates mitochondrial GSH transport. Knockdown of *SLC25A10* induces sensitivity to Gln deprivation, decreases NADP and NAPDH levels, and is associated with low expression of key genes involved in the antioxidant system including thioredoxin 2 (*TXN2*) and thioredoxin reductase 2 (*TXNRD2*). Consequently, the knockdown of *SLC25A10* increases sensitivity to oxidative stress and cisplatin treatment [110].

## 3. The Role of Amino Acid Transporters in the Epigenetic Regulation

**Figure 3:**
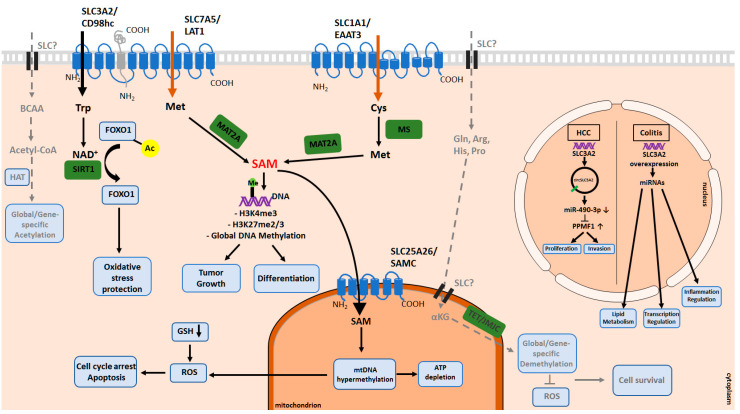
Epigenetic modifications controlled by amino acid transporters. Amino acid transporters play an important role in global or gene-specific epigenetic regulations. The LAT1(SLC7A5)/CD98hc (SLC3A2) complex is among the amino acid transporters responsible for Trp and Met uptake. SAM is produced from intracellular Met by MAT2A and is used as a methyl donor for methylation reactions. SLC1A1/EAAT3 is among the regulators of methylation reactions through Cys uptake, which is subsequently converted to Met by MS. SAM can be transported into mitochondria by SAMC, a mitochondrial carrier which was shown to be important for mtDNA hypermethylation and subsequently intracellular ROS levels. Intracellular tryptophan, which is transported by SLC7A5, is used for de novo NAD+ production and intracellular NAD+ levels control the activity of HDACs. In the presence of NAD+, SIRT1 removes the acetyl group and inactivates FOXO1, a transcription factor, and a critical cellular redox sensor protecting the cell from oxidative stress. In colitis, SLC3A2 overexpression results in increased miRNA-132 expression levels and consequently, downregulation of its target genes that are involved in different pathways, including lipid metabolism, transcriptional regulation and inflammation regulation. circSLC3A2, a circular RNA encoded by SLC3A2, induces proliferation and invasion in HCC by sponging miR-490-3p and regulating PPM1F expression. Amino acid transporters regulating the demethylation or acetylation reactions remain to be determined. Acetyl-CoA: Acetyl Coenzyme A; Ac: Acetylation; αKG: Alpha-ketoglutarate; Arg: Arginine; Cys: Cysteine; Gln: Glutamine; GSH: Glutathione; HAT: Histone Acetyltransferase; His: Histidine; Met: Methionine; MS: Methionine Synthase; NAD+: Nicotinamide Adenine Dinucleotide; Pro: Proline; ROS: Reactive Oxygen Species; SAM: S-Adenosyl methionine; TET: Ten-Eleven Translocation; Trp: Tryptophan.

Epigenetic regulation of amino acid transporters has been frequently shown in different cancer types [111,112,113,114]. Recently, the reciprocal regulation between amino acid transporters and epigenetic alterations has drawn attention. The altered metabolism reprograms the cancer epigenome, which is involved in cancer development and progression (Figure 3). To date, DNA methylation reactions and histone modifications are among the most broadly studied epigenetic alterations. These epigenetic signatures are dependent on specific regulators that require different metabolites as substrates and cofactors. The availability of most of these metabolites is dependent on amino acid transporter activity [115,116,117,118].

### 3.1. DNA and Histone Methylation and Demethylation Processes

The methylation reaction, a covalent addition of methyl group, is catalyzed by three DNA methyltransferase isoforms (DNMT1, DNMT3A, DNMT3B) and by various histone methyltransferases (HMTs). Methyl groups are added to cytosine nucleotides on DNA to form 5-methylcytosine at CpG locations (5mC), a well-known transcriptional repressive mark. Methylation of histone H3 and H4 at lysine (Lys) and Arg residues can be associated with either repressive or activating transcriptional effect, depending on the location and the number of methyl groups. All cellular methyltransferases require S-adenosylmethionine (SAM) as the universal methyl donor. SAM is produced from methionine (Met) by methionine adenosyltransferase (MAT) in the methionine cycle pathway [119]. Thus, alterations in the Met metabolism influencing the intracellular SAM levels can directly affect methylation levels and, subsequently, gene expression. The amino acid transporter SLC7A5/LAT1, with its obligate chaperone and dimerization partner SLC3A2/CD98hc, has been shown to have a high affinity for Met [120]. SLC7A5 and SLC3A2 expression is highly upregulated in many tumor types, such as HNSCC, prostate, lung, breast, and pancreatic cancers [49,121,122,123,124,125].

Gene knockdown experiments revealed a critical role of SLC7A5 for the transport of Met and maintenance of the intracellular SAM levels in lung cancer [126]. The reduction in the SLC7A5 levels decreases the activity of enhancer of zeste homolog 2 (EZH2) and levels of EZH2-dependent di- and tri-methylation of H3K27. The regulation between SLC7A5 and EZH2 is reciprocal, and EZH2 controls the expression levels of SLC7A5 through epigenetic inhibition of RXRa. Apart from the findings in lung cancer, a recent report showed that triggering naïve T cells via antigen receptors increases SLC7A5 expression and bioavailability of Met and results in increased levels of H3K4me3 and H3K27me3 in vitro and in vivo. These epigenetic alterations drive effector T lymphocyte differentiation [127,128]. The influence of amino acid transporters on epigenetic regulations has been shown not only on the nuclear DNA but also on the mitochondrial DNA. SLC25A26 gene encodes the mitochondrial carrier responsible for the import of SAM into the mitochondrial matrix. Menga et al. revealed that SLC25A26 is downregulated in cervical cancer due to promoter hypermethylation [113]. Overexpression of SLC25A26 increases the uptake of cytosolic SAM into the mitochondria. Methylation analyses showed that the mitochondrial transcription controller region, displacement loop (D-loop), is hypermethylated due to higher mitochondrial SAM levels. SLC25A26 overexpression is associated with hypermethylation of mitochondrial DNA, reduced oxidative phosphorylation, and, subsequently, lower mitochondrial ATP levels. Also, increased mitochondrial SAM availability reduces levels of GSH, leading to oxidative stress, cell cycle arrest, and apoptosis. Another study by Hodgson et al. showed that SLC1A1, which is responsible for Cys influx, is essential for redox balance. Blocking this transporter leads to oxidative stress, and methionine synthase (MS) inhibition subsequently decreases global DNA methylation levels in neuroblastoma cells [129]. Like methyltransferases, demethylases also require metabolites as substrates and cofactors to erase the methyl groups from DNA and histones. α-ketoglutarate (αKG) is one of the major metabolites utilized by demethylases. It can be produced from isocitrate in the citric acid cycle in mitochondria and also can be synthesized from different amino acids such as Gln, Arg, His, and proline (Pro) [130,131,132,133]. In glioma and cholangiocarcinoma, αKG can be a precursor for oncometabolite that leads to DNA and histone methylation aberrations resulting in stem-like phenotype switch [134,135,136]. Thus, the amino acid transporters responsible for intracellular Gln transport, as well as amino acids contributing to the αKG synthesis, can be potential epigenetic regulators through this metabolic pathway. However, the additional links between amino-acid transporters, DNA and histone demethylation processes, and their role in tumor development remain to be determined.

### 3.2. Histone Acetylation and Deacetylation Mechanisms

Histone acetylation is the addition of a negatively charged acetyl group to the Lys residues of the histone proteins. It subsequently reduces the binding between histones and DNA and induces the recruitment of transcriptional activators [137]. Histone acetylation and deacetylation are mediated by histone acetyltransferases (HAT) and histone deacetylases (HDAC), respectively. They are profoundly affected by cellular acetyl-coenzyme A (CoA) abundance, as a major acetylation source [115,138,139]. Acetyl-CoA can be derived from BCAAs (Leu, isoleucine (Ile), valine (Val) and Lys), which can be further utilized by HATs [140]. Transporters regulating the intracellular levels of these amino acids may play a role in controlling the Acetyl-CoA availability, therefore global or gene-specific acetylation levels.

On the other hand, deacetylation reactions require nicotinamide adenine dinucleotide (NAD+), an oxidizing agent. NAD+ serves as a cofactor for the class III HDAC enzymes known as sirtuins [141] and can be synthesized de novo from tryptophan (Trp). Therefore, intracellular NAD+ availability can regulate the catalytic activity of sirtuins, mostly SIRT1. In addition to histones, other epigenetic regulators such as FOXO1, a transcriptional factor, and a critical cellular redox sensor, can also be regulated by SIRT1-dependent deacetylation. In particular, studies in lung cancer showed that de novo synthesis of NAD+ from Trp is essential for resistance to oxidative stress [142]. The inhibition of SLC7A5 leads to the disruption of Trp uptake, depleting the NAD+ pool, and SIRT1 activity. Consequently, it leads to the accumulation of acetylated FOXO1, which is then translocated to the nucleus and activates downstream pro-apoptotic genes. This study shows that in tumor tissues, increased levels of SLC7A5 may represent an adaptation mechanism for resistance to oxidative stress in cancer cells through regulating epigenetic modifications. These findings reinforce the potential roles of amino acid transporters as metabolic signaling components in the regulation of the cellular epigenetic landscape. 

### 3.3. Other Chromatin Modifications and Epigenetic Regulations 

Apart from the epigenetic regulations described above, amino acid transporters influence different chromatin modifications, including the expression of miRNAs. In both in vitro and in vivo conditions, SLC3A2 is highly expressed during intestinal inflammation, which is a risk factor for developing colorectal cancer (CRC) [143,144]. SLC3A2 overexpression in intestinal epithelial cells under inflammatory conditions induces miRNA-132 expression. Consequently, it leads to the downregulation of the miRNA-132-target genes, which are involved in lipid metabolism and transcriptional regulation. Similar gene expression changes also occur in patients with inflammatory bowel disease [145]. Furthermore, by regulating colonic miRNAs, SLC3A2 may impact tight junction proteins, which play a role in colonic barrier dysfunction during intestinal colitis [146]. The study by Han et al. showed that SLC3A2 overexpression induces colon inflammation and increases the risk of colitis-associated cancer via dysregulating broad miRNA expression profile in both tissue villus and crypt cells [147]. Wang et al. revealed that SLC3A2-encoding circular RNA (circRNA) is a critical regulator of tumor growth. circRNA is a new class of non-coding RNAs that has a closed-loop structure and higher conservation between the species. circSLC3A2 regulates cellular proliferation and invasion in HCC by sponging miR-490-3p and upregulating its target, protein phosphatase, Mg2+/Mn2+ dependent 1F (PPM1F) [148]. This study shows that circSLC3A2 is a potential oncogenic factor in HCC, and by modulating downstream players, it may lead to tumor progression. Taken together, these studies demonstrate the possible importance of amino acid transporters for the progress of carcinogenesis through epigenetic modifications.

## 4. Amino Acid Transporters as Targets for Tumor Treatment

In contrast to normal tissues, many malignancies depend on exogenous supplies. Multiple tumor-promoting genetic mutations cause metabolic reprogramming to provide a growth advantage to highly proliferating cells over healthy cells. Thus, cancer cells upregulate different amino acid transporters to elevate the uptake of nutrients and sustain bioenergetic and biosynthetic processes. Therefore, the lack of certain nutrients due to the targeting of amino acid transporters may cause cancer cell death. On the other hand, normal cells under the same conditions have a better chance of surviving due to their less addiction to non-essential nutrients that can be synthesized from other metabolic intermediates. Unlike normal cells, for instance, acute lymphoblastic leukemia (ALL) cells cannot synthesize asparagine due to the lack of asparagine synthase (ASNS) expression, making them heavily dependent on exogenous asparagine to survive. Thus, L-asparaginase (ASNase) treatment in ALL patients catalyzes the conversion of plasma L-asparagine to aspartic acid and ammonia, leading to the death of cancer cells due to the inhibited global protein synthesis. Similarly, deterioration of asparagine uptake might also cause the death of ALL but not normal cells [149,150,151,152]. 

There are several strategies to alter the amino acid balance in cancer cells. One approach is to apply amino acid starvation to decrease cancer cell growth [153,154]. Secondly, some transporters might be recruited to selectively increase the concentration of specific amino acids [155,156,157]. Last but not least, transporters might be blocked to prevent amino acid influx or efflux [158,159,160], Table 1. The preclinical inhibition of Gln transporters, which contribute to the anti-oxidative cell defense, in particular, SLC1A5/ASCT2, SLC7A5/LAT1, and cystine/glutamate antiporter SLC7A11/xCT attracted the most attention and will be discussed in more detail. 

### 4.1. Targeting SLC1 Transporter Family

The expression of SLC1A5 is frequently upregulated in the different types of cancer with high Gln demand, and its increased expression is associated with worse clinical prognosis. The preclinical studies using in vitro and in vivo models demonstrated the importance of SLC1A5-mediated Gln uptake for tumor growth, making it an attractive therapeutic target [193]. Schulte et al. developed V-9032, an antagonist of transmembrane Gln flux. V-9032 specifically and effectively targets SLC1A5 and thus leads to the attenuation of proliferation and growth of cancer cells as well as elevation of oxidative stress and cell death, contributing to anti-tumor responses both in vivo and in vitro [163]. Furthermore, either short hairpin RNA (shRNA) or miR-137-mediated silencing of SLC1A5 and pharmacological inhibition of SLC38A2 and SLC1A5 by V-9302 abolish intracellular Gln levels as well as Gln metabolism that eventually affects GSH production [29]. Overall, these effects lead to an increase in apoptosis, autophagy, and oxidative stress and suppression of the mTORC1 signaling and cell proliferation in HNSCC, further boosting tumor cell sensitivity to cetuximab treatment [29]. Inhibition of SLC1A5 in CRC cells by shRNAs or L-g-glutamyl-p-nitroanilide (GPNA) reduces the growth of tumor cells, induces DNA damage, and augments the efficacy of cetuximab on cell proliferation [164]. Additionally, GPNA or benzylserine (BenSer)-mediated inhibition of SLC1A5 markedly reduces the growth of endometrial cancer cells, recapitulating the effect from SLC1A5 knockdown [165]. Also, pharmacological or genetic inhibition of SLC1A5 in NSCLC cells reduces Gln consumption, induces autophagy, and apoptosis as well as reduces tumor growth in NSCLC xenografts [194]. Bröer et al. tested the specificity of 2-amino-4-bis(aryloxybenzyl)aminobutanoic acids (AABA) group of inhibitors, compound 12 [167], and V-9302 [163], in human cancer cell lines with KO of SLC1A5 expression [34]. Interestingly, while these compounds suspend Gln transport in SLC1A5 KO cells but not in the wild type (WT) cells, SLC1A5 KO and WT cells exhibit equal sensitivity to these inhibitors [27]. Both amino acid ablation and SLC1A5 depletion augment SLC38A2 expression, although AABA compounds inhibit the activity of SLC38A2 [34]. Additionally, AABA compounds inhibit Ile uptake via SLC7A5 [34]. Bröer et al. demonstrated that compound 12 or V-9302 do not inhibit SLC1A5 but rather blocks SLC38A2 and SLC7A5, thereby resultant amino acid imbalance leads to the biological effects also described in other studies [34,163,167]. Treatment with an approved anti-epidermal growth factor receptor (EGFR) therapeutic antibody, cetuximab, may increase the susceptibility of HNSCC cells to oxidative stress by decreasing intracellular GSH levels because of reduced Gln uptake [195,196]. Incorporating cetuximab with oxidative therapy might be a useful strategy to eliminate cetuximab-resistant and EGFR-overexpressing cancers [195,196]. On the other hand, targeting SLC1A5 even without combination with cetuximab may be sufficient to sensitize HNSCC cells to ROS-induced apoptosis [29]. Hara et al. developed a monoclonal antibody (mAB) against the extracellular domain of SLC1A5 that decreases intracellular Glu transport, phosphorylation of AKT and ERK as well as abrogates KRAS-mutant tumor growth in athymic mice in vivo [170]. Kasai et al. assessed the anti-tumor potency of novel anti- SLC1A5 humanized KM8094 mAB in gastric cancer patient-derived xenografts (PDXs) [168]. KM8094 mAB blocks Gln uptake, induces oxidative stress and apoptosis, and suppresses the growth of gastric cancer cells [197]. By a cell-based screen, Suzuki et al. established several mABs against SLC1A5 called KM4008, KM4012, and KM4018 and demonstrated that these mABs abolish the Gln-dependent growth of WiDr CRC cells [169]. Moreover, Zhou et al. developed a novel glutamine-β-cyclodextrin (GLN-CD) to deliver doxorubicin into TNBC tumors through SLC1A5 [191]. The doxorubicin-GLN-CD complex engenders G2/M arrest and apoptosis in vitro and markedly accumulates in tumors in vivo with minimum toxicity in main organs compared to doxorubicin itself [198]. Additionally, GW501516, a nuclear receptor peroxisome proliferator-activated receptor δ (PPARδ) agonist, significantly elevates GLUT1, and SLC1A5 expression, thereby promoting tumor growth in several cancer cell lines [199]. On the other hand, Metformin inhibits tumor growth through the reduction of glucose and Gln flux by inhibition of GLUT1 and SLC1A5 [199]. Wang et al. characterized a Gln analog called polyglutamine (PGS) to deliver therapeutic agents to Gln-addicted cancer cells and showed the importance of SLC1A5 for the cellular internalization of the small interfering RNA (siRNA)-PGS complexes [192]. Delta-tocotrienol (δT) markedly inhibits Gln uptake and leads to the reduction of Glu, GSH, and some EAAs in A549 and H1299 NSCLC cell lines by targeting SLC1A5 and SLC7A5 [171]. Thus, inhibition by δT suspends Gln metabolism, cell proliferation, and mTOR pathway, as well as induces apoptosis through the downregulation of mTOR signaling [171]. Moreover, chemical inhibition of SLC7A5 by BCH does not inhibit melanoma cell growth, whereas SLC1A5 inhibition by BenSer reduces 2D and 3D cell proliferation and cell cycle progression. This effect of BenSer is attributed to the inhibited cyclin-dependent kinase 1 (CDK1) and ubiquitin Conjugating Enzyme E2 C (UBE2C) expression as a result of the reduced Gln and Leu transport and downregulated mTORC1 signaling [166].

Wu et al. identified 2-(furan-2-yl)-8-methyl-*N*-(o-tolyl)imidazo(1,2-a)pyridin-3-amine as selective inhibitor of another member of SLC1 family, EAAT3/ SLC1A1 [161]. Moreover, Cheng et al. developed a photoswitchable inhibitor, Azo-TFB-TBOA, against EAAT family of Glu transporters, which could be reversibly altered from high-affinity to low-affinity configuration depending on the excitation [162]. While Azo-TFB-TBOA selectively inhibits EAAT2/ SLC1A2 in the dark, exposure to the light at 350 nm alters the configuration of Azo-TFB-TBOA and decreases its affinity against SLC1A2 [162]. Combination of EAAT1/ SLC1A3 inhibition with asparaginase (ASNase) treatment results in cell cycle arrest or apoptosis associated with inhibition of TCA cycle, energy production, redox homeostasis, and biosynthesis of lipids and nucleotides [200]. Although inhibition of EAAT activity can be a promising approach to treat certain types of malignancies such as lung and gastric cancer [32,201], EAAT transporters play a critical role in the maintenance Glu homeostasis in the brain. Therefore EAAT inhibition can be potentially associated with neuronal toxicity [30]. To reduce extracellular Glu levels and thus a possibility of excitotoxicity, EAAT inhibition can be potentially combined with inhibition of SLC7A11/xCT antiporter exchanging intracellular glutamate for extracellular cystine [202]. 

### 4.2. Targeting SLC7 Transporter Family

The L-type amino acid transporters LAT1/SLC7A5 and xCT/SLC7A11 are overexpressed in a wide variety of human malignancies and emerged as promising therapeutic targets. Genetic or pharmaceutical inhibition of SLC7A5 leads to amino acid stress response via GCN2 and ATF4 activation and mTORC1 inhibition, causing a growth arrest of tumor cells in vitro and in vivo [59]. Of note, SLC3A2 KO cancer cells become more sensitive to SLC7A5 depletion [59], although the genetic silencing of SLC3A2 itself exhibits cell-context-dependent effects on tumor growth and mTORC1 signaling activation [49,59]. Based on the structure of known SLC7A5 inhibitor triiodothyronine (T3), Kongpracha et al. developed a series of SLC7A5 inhibitors. One of them, called SKN103 is an efficient inhibitor of the SLC7A5-mediated Leu transport [176]. Additionally, SKN103 inhibits mTOR activity and growth of cancer cells. This inhibitory effect is markedly enhanced in combination with cisplatin treatment [176]. Huttunen et al. developed a selective and slowly reversible inhibitor that reduces SLC7A5 -mediated Leu uptake and breast cancer cell growth and also markedly augments the inhibitory effect of cisplatin and bestatin on tumor cell viability [203]. This inhibitor has high metabolic stability and selectivity against SLC7A5, but can dissociate from the blood-brain barrier and cell surface, meaning that the inhibition may be reversed gradually [203]. Another SLC7A5 inhibitor called JPH203 was shown to have an anti-cancer effect in a variety of tumor models. JPH203 inhibits thymic carcinoma cell growth in vitro [204], constrains in vitro cell migration, invasion, and proliferation of renal cell carcinoma (RCC) cell lines [205], and suppresses anaplastic thyroid cancer (ATC) cell proliferation by abolishing mTOR signaling and deterring the cell cycle [177]. Cormeiras et al. showed that JPH203 circumvents mTORC1 activity, amino acid homeostasis, and proliferation of medulloblastoma cells without causing drug resistance after long-term usage [178]. Furthermore, combinational use of metformin and JPH203 hinders HNSCC cell proliferation in vitro and in vivo much more dramatically than the mono treatments [179]. JPH203 reduces the proliferation and viability of leukemic cells, triggers autophagy and apoptosis in vitro, and inhibits the growth of xenografted PTEN deficient cells in nude mice. Additionally, chemical SLC7A5 inhibition hampers mTORC1 and AKT activations, reduces c-MYC expression, and activates UPR [180].

Preclinical studies also suggest that SLC7A5 inhibition can be more toxic for tumor cells than for normal tissues. Yun et al. showed that JPH203 suspends the proliferation of oral carcinoma YD-38 cells in a time and dose-dependent manner. In contrast, it was far less toxic for normal human oral keratinocytes (NHOK) proliferation [206]. Furthermore, YD-38 cells possess a high LAT1 expression, whereas NHOK cells have a high LAT2 and a weak LAT1 expression, which underlines lower sensitivity of normal cells to JPH203 mediated inhibition of LAT1 [206]. Similarly, JPH203 exhibits selective cytotoxicity to medulloblastoma and low toxicity towards normal tissues [178].

SLC7A11 protein is another attractive target for anti-cancer therapy due to its role in the GSH synthesis. However, the sensitivity to the SLC7A11 inhibition can be altered by metabolic reprogramming. Therefore, Otsuki et al. applied a combination of SLC7A11 inhibitor SSZ with Oxyfedrine (OXY), a vasodilator, and a β adrenoreceptor agonist, to sensitize a panel of cancer cell lines to the ROS-induced therapy [182]. The combination of OXY and SSZ treatments enhances cytotoxic aldehyde 4-hydroxynonenal (4-HNE) accumulation and induces necrotic cell death in SSZ-resistant cancers in vitro and in vivo [182]. Additionally, inhibition of aldehyde dehydrogenase (ALDH) enzyme by OXY sensitizes cancer cells to GSH deficiency in response to radiotherapy in vitro [182]. Furthermore, chemical or genetic inhibition of SLC7A11 inhibits tumor growth in vivo. It triggers death in KRAS-mutant cancer cells, suggesting that SLC7A11 may be an Achilles’ heel for KRAS-mutant lung adenocarcinoma (LUAD) [183]. While SSZ leads to selective killing in a panel of KRAS-mutant LUAD in vitro and inhibits tumor growth in vivo, inhibition by HG106, a potent inhibitor of SLC7A11, notably reduces Cys uptake, GSH biosynthesis and demonstrates selective cytotoxicity to KRAS-mutant LUAD cells through augmenting ER-stress and oxidative stress-mediated cell death [183]. SSZ-mediated SLC7A11 inhibition efficiently reduces the NSCLC cell proliferation and invasion in vivo and in vitro [181]. SSZ impairs GSH synthesis, as well as augments intracellular αKG levels, mitochondrial metabolism, and ROS accumulation in CD44v^high^ undifferentiated stem-like HNSCC cells [184]. Besides, SSZ fine-tunes the CD44v9-SLC7A11 system to hamper the metastatic potential of bladder cancer. It increases cisplatin-induced cytotoxicity through inhibition of GSH levels and production of ROS [185]. A combination of cisplatin and SSZ leads to severe cytotoxicity in MBT-2V cells through upregulation of phospho-p38 MAPK and inhibition of CD44v9 [185]. Targeting SLC7A11 with SSZ induces apoptosis of primary effusion lymphoma (PEL) cells and precludes tumor progression in xenograft models [187]. SSZ-treated PEL cells deregulate genes involved in oxidative stress, cell death, and UPR [186]. All in all, these preclinical studies demonstrate that inhibition of amino acid transporters might open a new avenue for cancer treatment by inhibition of the oxidative cell defense and sensitizing tumor cells to the conventional therapies. 

## 5. Molecular Imaging by Using Amino Acid Transporters

Positron emission tomography (PET) imaging uses a radiolabeled glucose analog ^18^F-2-fluoro-2-deoxyglucose (^18^F-FDG) and is widely applied in clinics for detection, staging, and monitoring responsiveness to treatment for most tumor types based on high glucose uptake by tumor tissues. Despite a high sensitivity of the FPG PET, there are several drawbacks of ^18^F-FDG imaging. First, some malignant tumors with low glycolytic activity, such as early-stage PCa, cannot be detected by FDG uptake [9,207]. Second, ^18^F-FDG imaging in some cancer types such as brain tumors and renal carcinoma has a poor sensitivity due to low contrast between malignant and normal tissues [208,209]. Further, increased ^18^F-FDG accumulation has been shown for some normal tissues such as the brain, muscles, gastrointestinal tract, brown adipose tissues, inflammation or infection sites, etc. [210,211,212], resulting in false-positive findings. For this reason, alternative non-FDG PET imaging approaches are being established for precise and accurate detection of tumor tissues, including PET imaging with amino acid transporter-based radiotracers (Table 2). 

### 5.1. SLC1A5/ASCT2-Dependent Tumor Imaging

The N-(2-(^18^F)fluoropropionyl)-L-glutamate ((^18^F)FPGLU) is a promising amino acid tracer for PET imaging of tumors with a high expression of extracellular Glu transporter excitatory amino acid carrier 1 (EAAC1) such as glioma and lung adenocarcinoma [213]. A ^18^F labeled alanine trifluoroborate (^18^F-Ala-BF3) is a selective SLC1A5 marker for cancer imaging in the gastric cancer xenograft models. Compared to FDG-PET, ^18^F-Ala-BF3 based imaging gives a high tumor-to-background contrast and low accumulation in the inflammation tissues [214]. Preclinical evaluation of another SLC1A5 marker, 4-[^18^F]Fluoro-Gln in lung cancer xenograft models showed a tumor-specific accumulation compared to normal lung tissues. In contrast to ^18^F-FDG, 4-(^18^F)Fluoro-Gln uptake by tumor cells positively correlates with SLC1A5 expression [215]. High demand for Gln is a feature of the highly aggressive neuroblastoma, and 4-(^18^F)Fluoro-Gln has been shown to have higher tumor uptake and tumor-to-muscle ratio than ^18^F-FDG tracer [228]. 4-(^18^F)Fluoro-Gln uptake in neuroblastoma cells is mainly mediated by SLC1A5, and overexpression of SLC1A5 is associated with poor clinical prognosis [37]. A preclinical study using PCa cell lines showed that upregulation of SLC1A5 expression in response to dihydrotestosterone (DHT) treatment correlates with increased uptake of [^14^C]fluciclovine, a tracer for the imaging of PCa [216]. However, analysis of (^18^F)fluciclovine PET scans of hormone naïve PCa patients demonstrates that (^18^F)fluciclovine uptake does not correlate with SLC1A5 expression [217]. Given the emerging role of SLC1A5 as a promising cancer biomarker, SLC1A5-specific tracers could be further developed into precision PET imaging diagnostic tools. 

### 5.2. SLC3 and SLC7-Dependent Tumor Imaging

The levels of CD98hc/SLC3A2 and its light chain subunits are upregulated in tumors, and its high expression is associated with tumor progression and treatment resistance [49,229]. It makes these amino acid transporters an attractive target for tumor imaging. 

O-(2-(^18^F)fluoroethyl)-l-tyrosine, (^18^F)FET is one of the first and most widespread 18F labeled tracers for L-type amino acid transporters [230]. LAT1/SLC7A5 is one of the amino acid transporters mediating (^18^F)FET accumulation in tumor cells [221,230]. (^18^F)FET is employed for brain tumor imaging, and is a powerful tool to discriminate recurrent glioblastoma from the posttreatment brain changes and to predict overall survival in glioblastoma patients [231]. Verhoeven et al. reported 2-(^18^F)-2-fluoroethyl-l-phenylalanine, 2-(^18^F)FELP as a new SLC7A5-targeting PET tracer for glioblastoma that is predominantly transported by the SLC7A5 and can discriminate glioblastoma from the radiation necrosis even better than (^18^F)FET [219,220]. Nozaki et al. identified SLC7A5 specific PET tracer, (S)-2-amino-3-[3-(2-^18^F-fluoroethoxy)-4-iodophenyl]-2-methylpropanoic acid, or ^18^F-FIMP. ^18^F-FIMP shows better discrimination between glioblastoma tumor tissue and inflammation lesions in xenograft mice models than other PET probes such as ^18^F-FET, 11C-MET, and ^18^F-FDG [222]. Another SLC7A5-specific tracer, ^18^F-fluoro-phenylalaine (^18^F-FBPA), has low accumulation in the inflammatory regions and high uptake by tumor tissues in the glioma xenograft model [232]. Aoki et al. suggested that the total distribution of ^18^F-FBPA and ^18^F-FAMT in glioma and pancreatic tumor xenografts does not depend on the tumor blood flow but might reflect the high level of SLC7A5 expression in tumor tissues [223]. SLC7A5 has upregulated expression in recurrent brain metastases as compared to healthy brain tissues. The SLC7A5 overexpression is associated with high uptake of (^18^F)-FDOPA, whereas radionecrosis regions with low SLC7A5 expression are characterized by an absence of (^18^F)-FDOPA signal [226]. The level of SLC7A5 expression significantly correlates with (^18^F)fluciclovine uptake by tumor tissues in patients with hormone naïve PCa, and tumors with high Gleason grade have higher SLC7A5 expression and ^18^F-fluciclovine signal [217].

In addition to the SLC7A5-specific radiotracers, fluorescent tracers are also under development. Matsuura et al. synthesized fluorescent polymer probes based on poly(N-isopropylacrylamide-co-N,N-dimethylacrylamide) conjugated to Tyr. This probe is specifically recognized by SLC7A5 and can be accumulated in cells in response to temperature increase, which improves probe hydrophilicity [225].

The probes specific for SLC3A2 imaging are also of high clinical importance. Deuschle et al. developed a high-affinity anticalin protein against human SLC3A2 ectodomain using phage display selection and protein design. ^89^Zr-labeled anticalin protein is characterized by strong and tumor-specific signals in PET imaging of xenograft tumor models for Burkitt′s lymphoma and metastatic PCa [218].

## 6. Conclusions

Amino acid transporters play a multifaceted role in tumor initiation, progression, and therapy resistance. They are critical to cover the energetic and biosynthetic demands of fast-growing tumors associated with high proliferation rates and nutrient-poor environments. Many amino acid transporters are highly expressed in tumors compared to the adjacent normal tissues, and their expression correlates with tumor progression, clinical outcome, and treatment resistance [57,154]. The deregulated levels of transporter proteins may be caused by frequent mutations of oncogenes and tumor suppressors. The transcription factor MYC, one of the most frequently amplified genes in human malignancies [233], is a master-regulator of amino acid metabolism promoting the expression of transporter proteins such as SLC1A5, SLC7A5 and SLC43A1 and thus, driving cancer growth by effective amino acid delivery [36,234,235]. In some tumors like PCa, upregulation and the interplay between MYC, AR, and the mTOR mutations cause Gln addiction when Gln becomes a conditional EAA for tumor growth and survival. These oncogenes increase Gln uptake by upregulation of SLC1A4 and SLC1A5 transporters [27]. 

A high expression of amino acid transporters in tumor cells is also critical for maintaining tumor redox homeostasis by regulating the intracellular GSH levels, ER stress, UPR signaling, and mTOR-mediated antioxidant defense. Amino acid transporters also play a critical role in the epigenetic adaptations of tumor cells to oxidative stress. These findings highlight the role of acid transporters as the defender of tumor antioxidant systems and genome integrity. 

Although functional characterization of amino acid transporters in different human malignancies received considerable attention in the last years, research on their potential employment as molecular biomarkers, therapeutic targets, and tools for tumor imaging is still in its infancy. Improvement in therapeutic targeting of amino acid transporters will require a better understanding of their plasticity and redundancy in the individual tumors. Recent studies showed that tumor cells dynamically upregulate redundant amino acid transporters and rapidly harmonize the intracellular amino acid pool in response to the amino acid deficiency in the tumor environment or transporter inhibition. Therefore, tumor cells with reduced transporter plasticity may be more sensitive to the inhibition of amino acid transporters than cells, which can quickly compensate for the ablation of transporters [42].

Another challenge for the therapeutic targeting of amino acid transporters is the activation of the compensatory pro-survival mechanisms. In response to the amino acid depletion, many tumors activate autophagy to overcome nutrient stress. Cell death and sensitivity to conventional treatment in response to the inhibition of amino acid uptake was substantially enhanced in autophagy-deficient tumor cells [49,236,237]. The ongoing clinical trials combine autophagy inhibition with chemotherapy or radiotherapy in patients with solid tumors. However, the role of autophagy as a tumor target is still debatable as it might promote or suppress tumor growth depending on tumor type and stage of tumor development [57,238].

Additionally, the affinity between amino acids and their transporters, availability of the amino acids, transporter expression levels, and redox homeostasis can be substantially influenced by microenvironmental conditions such as hypoxia, acidosis, and reactive stroma [239,240,241]. The research efforts over the past decade have advanced the experimental models for the analysis of tumor metabolism to recapitulate these microenvironmental factors, including patient tumor-derived organoid co-cultures, sliced tissue models, and in vivo stable isotope labeling [242,243,244].

While many potential preclinical approaches to inhibit amino acid transporters are available, none of them entered clinical trials. The development of novel metabolic drug targets requires multidisciplinary approaches to understand the physiological role and regulatory modes for the individual amino acid transporters in the different tumor and normal tissues. It includes multi-omics analyses in response to the transporter ablation combining genome, proteome, and metabolome levels, followed by data integration and computational modeling. Availability of the X-ray structures facilitates the identification of novel transporter inhibitors by structure-based design approach and virtual screening of millions of chemical molecules. Large-scale analysis of the patients’ omics data might help to elucidate the function of the individual transporters in cancer progression [242,245,246]. Finally, recent technological advances in the study of cancer metabolism that help to accurately detect metabolites in high-throughput scale with spatial and temporal resolution [242] and further development of the experimental models, which more accurately recapitulate tumor microenvironment, will play a pivotal role in the development of new efficient drugs and specific tracers toward amino acid transporters.

## Figures and Tables

**Table 1:** Targeting of the amino acid transporters.

Chemical Inhibition					
Transporter	Inhibitor	Experimental Model	Tissue Type		Refs
SLC1A1	2-(furan-2-yl)-8-methyl-*N*-(o-tolyl)imidazo[1,2-a]pyridin-3-amine	In vitro (Cell line)	Human Embryonic Kidney cells		[161]
SLC1A2	Azo-TFB-TBOA	In vitro (*Xenopus laevis* oocytes)	*Xenopus laevis* oocytes		[162]
SLC1A5, SLC38A2, SLC7A5	V-9302	In vitro (Cell line)	A panel of human cancer cell lines [163] Head and Neck Squamous Cell Carcinoma [29]		[29,163]
		In vivo (Cell line derived-xenograft)	patient-derived xenografts (PDX) model [163], Head and Neck Squamous Cell Carcinoma cell line xenograft [29]		
SLC1A5	L-g-glutamyl-p-nitroanilide (GPNA)	In vitro (Cell line)	Colorectal Carcinoma [164], Endometrial Carcinoma [165]		[164,165]
		In vivo (Cell line derived-xenograft)			
SLC1A5	Benzylserine (BenSer)	In vitro (Cell line)	Endometrial Carcinoma [165], Melanoma [166]		[165,166]
SLC1A5	Benzyloxybenzyl analogues	In vitro (Cell line)	Human Embryonic Kidney cells, C6 Rat cells		[167]
SLC1A5	KM8094 mAB	In vivo (Patient derived-xenograft)	Patient-derived Gastric cancer tissue		[168]
SLC1A5	KM4008 mAB	In vitro (Cell line)	A panel of cell lines		[169]
SLC1A5	KM4012 mAB	In vitro (Cell line)	A panel of cell lines		[169]
SLC1A5	KM4018 mAB	In vitro (Cell line)	A panel of cell lines		[169]
SLC1A5	Ab3-8 mAb	In vitro (Cell line)	Colorectal Carcinoma		[170]
SLC1A5, SLC7A5	Delta-tocotrienol (δT)	In vitro (Cell line)	Non-Small Cell Lung Cancer		[171]
SLC6A1	Photoswitchable inhibitor	In vitro (Cell line)	Human Embryonic Kidney cells and Dentate gyrus granule cells		[172]
SLC6A14	α-methyltryptophan	In vitro (Cell line)	Colon cancer [173], Pancreatic cancer [174,175]		[173,174,175]
		In vivo (Cell line derived-xenograft)			
SLC7A5	SKN103	In vitro (Cell line)	A panel of human cancer cell lines		[176]
SLC7A5	BCH	In vitro (Cell line)	Melanoma		[166]
SLC7A5	JPH203	In vitro (Cell line)	Anaplastic thyroid cancer [177], Medulloblastoma [178], Head and Neck Squamous Cell Carcinoma [179], T-cell lymphoblastic lymphoma/T-cell acute lymphoblastic leukemia [180]		[177,178,179,180]
		In vivo (Cell line derived-xenograft)			
SLC7A11	Sulfasalazine (SSZ)	In vitro (Cell line)	Non-Small Cell Lung Cancer [181], A panel of human cancer cell lines [182], Lung Adenocarcinoma [183], Head and Neck Squamous Cell Carcinoma [184], Bladder Cancer [185], Lymphoma [186,187]		[181,182,183,184,185,186,187]
		In vivo (Cell line derived-xenograft)			
		In vivo (KRAS Mouse model)			
		In vivo (Murine metastasis model)			
SLC25A8	Genipin	In vitro (Cell line)	Breast Cancer [188], Ovarian Cancer [189]		[189]
SLC43A1	ESK246	In vitro (Cell line)	Prostate Cancer		[190]
**Drug Delivery**					
**Through which Transporter**	**Molecule**	**Delivery**	**Experimental Model**	**Tissue Type**	**Refs**
SLC1A5	GLN-CD (conjugation of Gln with β-cyclodextrin)	Doxorubicin	In vitro (Cell line)	Triple-negative Breast Cancer	[191]
SLC1A5	Polyglutamine (PGS)	various agents including siRNAs	In vitro (Cell line)	Lung Cancer	[192]

**Table 2:** Molecular imaging by using amino acid transporters.

Transporter	Tracer	Experimental Model	Tissue Type	References
SLC1A1	*N*-(2-(^18^F)fluoropropionyl)-L-glutamate ((^18^F)FPGLU)	In vitro (Cell line)	Rat C6 glioma cell line, SPC-A-1 lung adenocarcinoma cell line	[213]
		In vivo (Cell line derived-xenograft)		
SLC1A5	^18^F-Ala-BF_3_	In vivo (Cell line derived-xenograft)	Gastric cancer cell line xenograft	[214]
SLC1A5	4-(^18^F)Fluoro-Gln	In vivo (Cell line derived-xenograft)	Non-Small Cell Lung Cancer and Colorectal Carcinoma xenografts	[215]
		In vivo (EGFR-mutant Mouse model)		
SLC1A5, SLC7A5	(^18^F)fluciclovine	In vitro (Cell line)	Castration-resistant prostate cancer	[216,217]
SLC3A2	Anticalin	Primary prostate cancer tissue	Primary prostate cancer tissue	[218]
SLC7A5	2-(^18^F)FELP	In vitro (Cell line)	Glioblastoma [219,220]	[219,220]
		In vivo (Cell line derived-xenograft)		
SLC7A5	[^18^F]FET	In vitro (Cell line)	Glioblastoma [219,220,221]	[219,220,221]
		In vivo (Cell line derived-xenograft)		
SLC7A5	^18^F-FIMP	In vivo (Cell line derived-xenograft)	Glioblastoma	[222]
SLC7A5	^18^F-FBPA	In vivo (Cell line derived-xenograft)	C6 Glioma and MIA PaCa-2 xenografts	[223]
SLC7A5	^18^F-FAMT	In vitro (Cell line)	C6 Glioma and MIA PaCa-2 xenografts [223], Xenopus oocytes [224]	[223,224]
		In vivo (Cell line derived-xenograft)		
		In vitro (*Xenopus laevis* oocytes)		
SLC7A5	P(NIPAAm-co-DMAAm)	In vitro (Cell line)	Cervical carcinoma (HeLa cells)	[225]
SLC7A5	(^18^F)-FDOPA	In vitro (Cell line)	Non tumoral brain tissues and brain metastases [226], Glioblastoma [227]	[226,227]
		Brain tumor tissue		

## Abbreviations

4-HNE	4-hydroxynonenal
4EBP1	Eukaryotic translation initiation factor 4E-binding protein 1
5mC	5-methylcytosine
18F-Ala-BF3	18F labeled alanine trifluoroborate
18F-FBPA	18F-fluoro-phenylalaine
18F-FDG PET	(18F)-Fluorodeoxyglucose Positron Emission Tomography
αKG	α-ketoglutarate
AA	Amino acid
AAA	Aromatic amino acid
AABA	2-amino-4-bis(aryloxybenzyl)aminobutanoic acid
AARE	Amino acid response element
Acetyl-CoA	Acetyl-Coenzyme A
Ac	Acetylation
AKT	AKT Serine/Threonine Kinase
ALDH	Aldehyde dehydrogenase
ALL	Acute lymphoblastic leukemia
AMPK	AMP-activated protein kinase
AR	Androgen Receptor
ARE	Antioxidant response element
Arg	Arginine
ASCT2/ SLC1A5	Alanine, serine, cysteine-preferring transporter 2
ASNase	Asparaginase
ASNS	asparagine synthase
Asp	Aspartate
ATC	Anaplastic thyroid cancer
ATF4	Activating Transcription Factor 4
ATF6	Activating Transcription Factor 6
ATG5	Autophagy Related 5
BCAA	Branched-chain amino acid
BenSer	Benzylserine
BSO	Buthionine sulphoximine
BTA	1,2,3-benzene-tricarboxylic acid
CAT	Catalase
CD44	Cluster of differentiation 44
CD44v	Cluster of differentiation 44 variant
CDK1	Cycling-Dependent Kinase 1
CDK7	Cyclin-Dependent Kinase 7
CIC	Citrate carrier
circRNA	Circular RNA
COX	Cyclooxygenase
CRC	Colorectal cancer
CSC	Cancer stem cells
Cys	Cysteine
δT	Delta-tocotrienol
D-loop	Displacement loop
DNA	Deoxyribonucleic Acid
DNMT	DNA Methyltransferase
EAA	Essential amino acid
EAAC1	Excitatory Amino Acid Carrier 1
EGFR	Epidermal Growth Factor Receptor
eIF2α	Eukaryotic Translation Initiation Factor 2A
ER	Endoplasmic reticulum
ERK	Extracellular signal-regulated kinase
Erα	Estrogen receptor alpha
ETC	Electron transport chain
EZH2	Enhancer of zeste homolog 2
GCL	Glutamate-cysteine ligase
GCN2	General amino acid control non-derepressible 2
Gln	Glutamine
GLN-CD	Glutamine-β-cyclodextrin
GSL	Glutaminase
Gly	Glycine
GPNA	l-g-glutamyl-p-nitroanilide
GPXs	Glutathione Peroxidases
GPX4	Glutathione Peroxidase 4
GSH	Glutathione
GSR	Glutathione reductase
GSS	Glutathione synthetase
GSSG	Glutathione disulfide
H_2_O_2_	Hydrogen Peroxide
HAT	Histone Acetyltransferase
HATC	Heterodimeric transmembrane amino acid transporter complex
HCC	Hepatocellular Carcinoma
HDAC	Histone Deacetylase
HIF1α	Hypoxia Inducible Factor 1 Subunit Alpha
HIF2α	Hypoxia Inducible Factor 2 Subunit Alpha
His	Histidine
HMT	Histone Methyltransferase
HNSCC	Head and neck squamous cell carcinoma
Ile	Isoleucine
IMCA	2-imino-6-methoxy-2H-chromene-3-carbothioamide
IR	Ionizing radiation
ISR	Integrated Stress Response
JDP2	Jun Dimerization Protein 2
KDM3B	Lysine Demethylase 3B
KO	Knockout
KRAS	KRAS Proto-Oncogene, GTPase
LAT1/SLC7A5	L-type Amino Acid Transporter 1
Leu	Leucine
lncRNA-XIST	Long Non-Coding RNA (lncRNA) X-Inactive Specific Transcript (XIST)
LOX	Lipoxygenase
LUAD	Lung adenocarcinoma
Lys	Lysine
mAB	monoclonal antibody
MAPK	Mitogen-activated protein kinase
MAT	Methionine Adenosyltransferase
MDR1	Multidrug resistance protein 1
Met	Methionine
miRNA	MicroRNA
MS	Methionine Synthase
mTOR	Mammalian target of rapamycin
mTORC1	Mammalian target of rapamycin complex 1
mTORC2	Mammalian target of rapamycin complex 2
NAC	*N*-acetyl-l-Cysteine
NAD+	Nicotinamide Adenine Dinucleotide
NADPH	Nicotinamide Adenine Dinucleotide Phosphate
NF-kB	Nuclear Factor kappa B
NOX	NADPH Oxidase
Nrf2	Nuclear factor-erythroid 2 -related factor 2
OXPHOS	Oxidative Phosphorylation
OXY	Oxyfedrine
PEL	Primary Effusion Lymphoma
P-gp	P-glycoprotein
p70S6k	The 70-kDa ribosomal protein S6 kinase
PCa	Prostate Cancer
PDH	Pyruvate dehydogenase
PDX	Patient-derived xenograft
PET	Positron emission tomography
PGS	Polyglutamine
PP2Ac	Protein Phosphatase 2 Catalytic Subunit Alpha
PPARδ	Peroxisome Proliferator Activated Receptor Delta
PPM1F	Protein phosphatase, Mg2+/Mn2+ dependent 1F
Pro	Proline
PRX	Peroxiredoxin
RB	Retinoblastoma protein
RNA	Ribonucleic acid
RCC	Renal Cell Carcinoma
RONS	Reactive oxygen and nitrogen species
ROS	Reactive oxygen species
S6K1	The mTOR Substrate S6 Kinase 1
SAM	S-Adenosylmethionine
Ser26	Serine26
shRNA	Short hairpin RNA
siRNA	Small interfering RNA
SLC	Solute carrier
SLC1A1	Solute Carrier Family 1 Member 1
SLC1A2	Solute Carrier Family 1 Member 2
SLC1A3	Solute Carrier Family 1 Member 3
SLC1A4	Solute Carrier Family 1 Member 4
SLC1A5	Solute Carrier Family 1 Member 5
SLC1A6	Solute Carrier Family 1 Member 6
SLC1A7	Solute Carrier Family 1 Member 7
SLC25A1	Solute Carrier Family 25 Member 1
SLC25A4	Solute Carrier Family 25 Member 4
SLC25A5	Solute Carrier Family 25 Member 5
SLC25A8	Solute Carrier Family 25 Member 8
SLC25A10	Solute Carrier Family 25 Member 10
SLC25A23	Solute Carrier Family 25 Member 23
SLC25A26	Solute Carrier Family 25 Member 26
SLC25A32	Solute Carrier Family 25 Member 32
SLC38A1	Solute Carrier Family 38 Member 1
SLC38A2	Solute Carrier Family 38 Member 2
SLC38A3	Solute Carrier Family 38 Member 3
SLC3A1	Solute Carrier Family 3 Member 1
SLC3A2	Solute Carrier Family 3 Member 2
SLC6A1	Solute Carrier Family 6 Member 1
SLC6A14	Solute Carrier Family 6 Member 14
SLC7A1	Solute Carrier Family 7 Member 1
SLC7A2	Solute Carrier Family 7 Member 2
SLC7A3	Solute Carrier Family 7 Member 3
SLC7A4	Solute Carrier Family 7 Member 4
SLC7A5	Solute Carrier Family 7 Member 5
SLC7A6	Solute Carrier Family 7 Member 6
SLC7A7	Solute Carrier Family 7 Member 7
SLC7A8	Solute Carrier Family 7 Member 8
SLC7A9	Solute Carrier Family 7 Member 9
SLC7A10	Solute Carrier Family 7 Member 10
SLC7A11	Solute Carrier Family 7 Member 11
SLC7A12	Solute Carrier Family 7 Member 12
SLC7A13	Solute Carrier Family 7 Member 13
SLC7A14	Solute Carrier Family 7 Member 14
SLC7A15	Solute Carrier Family 7 Member 15
SNAT1/ SLC38A1	Sodium-coupled neutral amino acid transporter 1
SNAT2/SLC38A2	Sodium-coupled neutral amino acid transporter 2
SOD	superoxide dismutase
SorCS2	Sortilin Related VPS10 Domain Containing Receptor 2
SSZ	Sulfasalazine
T3	Triiodothyronine
TCA cycle	Tricarboxylic acid cycle
TET	Ten-Eleven Translocation
TMZ	Temozolomide
TNBC	Triple-negative breast cancer
Trp	Tryptophan
TRX	Thioredoxin
TXN2	Thioredoxin 2
TXNRD	Thioredoxin Reductase
UBE2C	Ubiquitin Conjugating Enzyme E2 C
UPR	Unfolded protein response
UPS	Ubiquitin-Proteasome System
Val	Valine
VPS10	Vacuolar protein sorting 10
VSMC	Vascular smooth muscle cells
WT	Wild type
XO	Xanthine oxidase
